# Calcitriol Supplementation Causes Decreases in Tumorigenic Proteins and Different Proteomic and Metabolomic Signatures in Right versus Left-Sided Colon Cancer

**DOI:** 10.3390/metabo8010005

**Published:** 2018-01-11

**Authors:** Monica M. Schroll, Katelyn R. Ludwig, Kerry M. Bauer, Amanda B. Hummon

**Affiliations:** Department of Chemistry and Biochemistry, Harper Cancer Research Institute, University of Notre Dame, Notre Dame, IN 46556, USA; Monica.M.Schroll.1@nd.edu (M.M.S.); kludwig1@nd.edu (K.R.L.); kbauer125@gmail.com (K.M.B.)

**Keywords:** vitamin D, calcitriol, colon cancer, proteomics, metabolomics, iTRAQ

## Abstract

Vitamin D deficiency is a common problem worldwide. In particular, it is an issue in the Northern Hemisphere where UVB radiation does not penetrate the atmosphere as readily. There is a correlation between vitamin D deficiency and colorectal cancer incidence and mortality. Furthermore, there is strong evidence that cancer of the ascending (right side) colon is different from cancer of the descending (left side) colon in terms of prognosis, tumor differentiation, and polyp type, as well as at the molecular level. Right-side tumors have elevated Wnt signaling and are more likely to relapse, whereas left-side tumors have reduced expression of tumor suppressor genes. This study seeks to understand both the proteomic and metabolomic changes resulting from treatment of the active metabolite of vitamin D, calcitriol, in right-sided and left-sided colon cancer. Our results show that left-sided colon cancer treated with calcitriol has a substantially greater number of changes in both the proteome and the metabolome than right-sided colon cancer. We found that calcitriol treatment in both right-sided and left-sided colon cancer causes a downregulation of ribosomal protein L37 and protein S100A10. Both of these proteins are heavily involved in tumorigenesis, suggesting a possible mechanism for the correlation between low vitamin D levels and colon cancer.

## 1. Introduction

**Figure 1:**
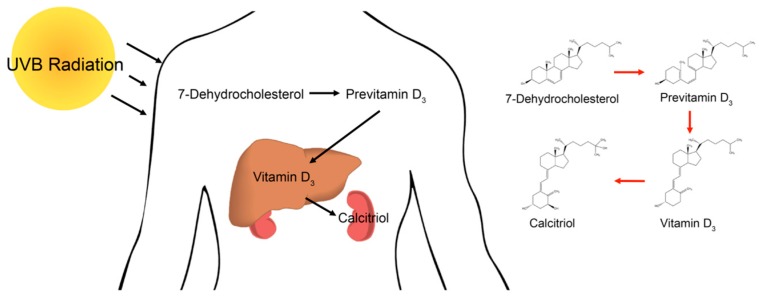
Illustration showing the conversion of 7-dehydrocholesterol into the active metabolite calcitriol in human liver and kidneys.

In humans, vitamin D is obtained through diet or is produced when the skin is exposed to ultraviolet light (Figure 1) [1]. Vitamin D deficiency is a common problem in the Northern Hemisphere where UVB rays from the sun do not readily penetrate the atmosphere for many months of the year. The active metabolite of vitamin D is 1,25-dihydroxyvitamin D (1α,25(OH)_2_D_3_), which is also known as calcitriol. Calcitriol is derived from vitamin D through a multistep process in the liver and kidneys [2]. This process begins when UVB radiation penetrates the skin and converts 7-dehydrocholesterol into previtamin D_3_. Previtamin D_3_ is then isomerized into vitamin D_3_, which enters the liver and kidneys and is metabolized into calcitriol (Figure 1). This active metabolite, calcitriol, acts as a steroid hormone and binds to the vitamin D receptor (VDR). Once the VDR is activated, it will bind to the vitamin D response elements (VDREs) on target genes, causing recruitment of either activators or inhibitors to change gene expression [3]. 

Vitamin D is vital for bone formation and mineralization, insulin secretion, and immunity. Vitamin D deficiency is also implicated in increased cancer risk [2,4]. It has been reported that since 1980 in the United States, there is a higher rate of colorectal cancer in the northeast and a lower incidence in the west and southwest [5,6]. This trend spurred investigations into the role of vitamin D in colorectal cancer [7]. Low serum levels of vitamin D have been linked to an increase in many solid tumor cancers including breast, prostate, and colorectal [1,8,9]. Vitamin D treatment has been shown to have chemopreventative activity in many different cancer types as reviewed by Giammanco et al. [8,10]. Specifically, vitamin D treatment in many solid tumor studies has been shown to have anti-inflammatory, anti-growth, and pro-differentiation effects [10]. We are interested in vitamin D supplementation and the effects it has on the proteome and metabolome of colon cancer development and progression.

**Figure 2:**
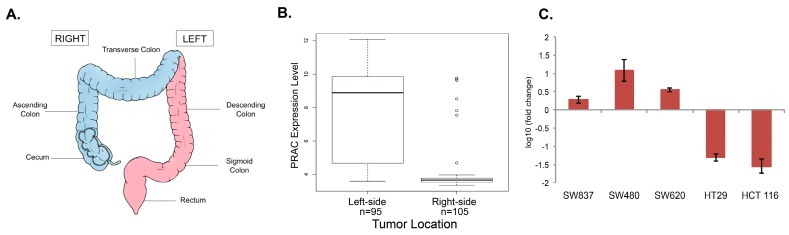
(**A**) Illustration of the human colon and rectum to denote right-sided colon cancer (RCC) from left-sided colon cancer (LCC); (**B**) Expression levels of the prostate cancer susceptibility candidate (PRAC) (230784_at) by tumor location in the GSE14333 microarray data. PRAC is expressed at a high level in most left-sided tumors and at a baseline level in most right-sided tumors; (**C**) qRT-PCR of PRAC expression in DLD-1, SW837, SW480, SW620, HT29, and HT 116 cells. PRAC mRNA levels were quantified using the comparative C_T_ method relative to mRNA levels of hypoxanthine phosphoribosyltransferase 1 (HPRT1). The fold change in expression (log_10_) was normalized to DLD-1 mRNA levels and is shown in the bar graph with the error bars representing the standard deviation.

The human colon has two sides; a right or proximal side, containing the ascending and transverse colon, and the left or distal side, which is comprised of the descending and sigmoidal colon (Figure 2A) [11,12,13,14,15]. The splenic flexure denotes the boundary between the two sides of the colon. During embryogenesis, the two sides of the colon originate from the midgut and the hindgut, respectively. Cancerous lesions can develop on either side of the colon and metadata analysis has confirmed that there are both phenotypic and molecular differences between the cancers that plague the left and right sides of the colon. Some of these differences include incidence: one-third of all colon cancers occur on the right side and two-thirds occur on the left side [11]. Right-sided colon cancer (RCC) is more prominent in women and left-sided colon cancer (LCC) is more common in men [12]. At the molecular level, a significant number of genes are differentially expressed in LCC and RCC, and the patterns of loss of heterozygosity and promoter methylation vary between locations [16,17]. In addition, LCC patients with KRAS mutations have a poorer prognosis, but KRAS mutation status does not have an effect on the prognosis in RCC patients [18]. Finally, LCC has a better prognosis than RCC, although the mechanism that explains the prognostic difference is unknown [12,13,14,15]. 

In this study, we treated two different cell lines derived from right-sided and left-sided colon cancer patients with calcitriol and examined the proteomic and metabolomic changes that occurred on the different sides of the colon. The first cell line is HCT 116, which is derived from a right-sided carcinoma, while the second is DLD-1, which was derived from a left-sided colon lesion. Since these cells are not capable of metabolizing 7-dehydrocholesterol into calcitriol, the cells were treated directly with calcitriol, the active metabolite of vitamin D. We employed ultra-high performance liquid chromatography–tandem mass spectrometry (UPLC-MS/MS) to survey and quantify both the proteins and metabolites in these cell lines after treatment with calcitriol. Proteins were digested with the enzyme trypsin into their corresponding peptides, separated, and analyzed by a mass spectrometer using optimized methods to examine both the proteome and the metabolome. 

Our findings indicate that calcitriol treatment caused distinct proteomic and metabolomic changes in RCC compared to LCC. We determined that more proteins and metabolites were altered in expression in LCC when treated with calcitriol. In particular, proteins and metabolites that regulate cholesterol biosynthesis pathways were significantly altered. Additionally, only two differentially regulated proteins overlapped when LCC and RCC were treated with calcitriol. One of these proteins, S100A10, is a known target for cancer treatment as it has a role in promoting angiogenesis [19]. Calcitriol treatment caused a downregulation of this protein in both RCC and LCC, which supports the role of vitamin D supplementation as a tool in cancer prevention and treatment. This study further supports the growing evidence that LCC and RCC should be studied as separate diseases and that they follow separate mechanisms to tumorigenesis. 

## 2. Results

### 2.1. Analysis of PRAC Expression in Human Colorectal Cancer Cell Lines to Determine Tissue Origin

There are numerous colorectal cancer cell lines available for research. However, with the exception of the DLD-1 cell line, the location of the tissue source within the colon is often not reported. The original manuscript describing the development of the DLD-1 cell line explicitly states that the tissue sample was derived from the left side of the colon [20]. These cells were collected from a 45-year-old male diagnosed with Duke’s C adenocarcinoma of the sigmoid colon [20]. 

Prostate cancer susceptibility candidate (PRAC) is a gene that is expressed only in the prostate, distal colon, and rectum [21]. Using this information, we hypothesized that PRAC expression could be used to determine the origin of the colon cancer tumor. We examined the expression of PRAC in 95 samples of LCC and 102 samples of RCC from a GSE14333 microarray dataset from the Gene Expression Omnibus. PRAC is significantly highly expressed in left-sided tumors and either expressed at a low baseline level or not detectable by microarray analysis in right-sided tumors (Student’s *t*-test *p*-value 3.677 × 10^−13^) (Figure 2B). This data provided statistically significant evidence to use PRAC expression as a measure to predict the sidedness of colorectal cancer cell lines. 

The mRNA expression level of PRAC, as measured by quantitative reverse transcriptase polymerase chain reaction (qRT-PCR), was measured in a panel of colon cancer cell lines derived from unknown tumor locations within the human colon. The PRAC mRNA expression levels in these cell lines were compared to the expression level in DLD-1, a cell line known to derive from a tumor in the distal colon, to elucidate the origin of the cell line material as ascending or distal colon. SW837, SW480, and SW620 have higher PRAC expression levels than DLD-1, while HT-29 and HCT 116 have almost non-detectable PRAC expression levels (Figure 2C). Based on these gene expression data patterns, we concluded that DLD-1, SW480, and SW620 were derived from material collected on the left side of the colon (LCC) and HT-29 and HCT 116 were from the right side (RCC). Using these results, we paired DLD-1 (LCC) and HCT 116 (RCC) cell lines together to study the differential response to vitamin D supplementation. 

The DLD-1 and HCT 116 cell lines share many critical similarities; both were obtained from male patients, are diploid in origin, are microsatellite unstable (MSI), and are KRAS mutant [22]. Thus, while they were derived from distinct areas of the colon, they do not have significant ploidy differences in their genomes. 

### 2.2. Proteomic Analysis of Differentially Regulated Proteins in Response to Calcitriol Treatment in RCC and LCC

**Figure 3:**
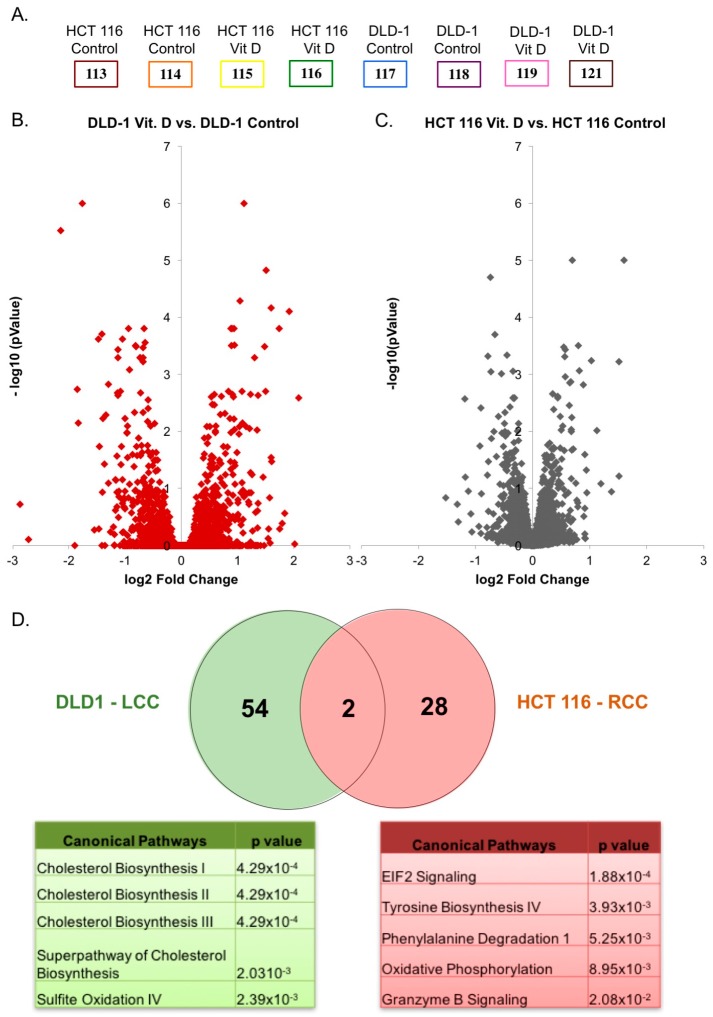
(**A**) Experimental setup of iTRAQ 8-plex experiment for quantitative proteomics. Each biological condition was labeled in duplicate; (**B**) Volcano plot of proteomic data (proteins were ranked according to their *p*-value (y-axis) and their relative abundance ratio (Log2 fold change) between DLD-1 treated vs. control (x-axis)). Proteins with a *p*-value of <0.01 and log2 fold change ± 1.5 were considered differentially regulated; (**C**) Volcano plot of proteomic data (proteins were ranked according to their *p*-value (y-axis) and their relative abundance ratio (Log2 fold change) between HCT 116 treated vs. control (x-axis)). Proteins with a *p*-value of <0.01 and log2 fold change ± 1.5 were considered differentially regulated; (**D**) Venn diagram showing overlap between differentially regulated proteins of DLD-1 and HCT 116 cells treated with calcitriol. Ingenuity Pathway Analysis was used to look at the canonical pathways enriched due to differentially regulated proteins in DLD-1 and HCT 116 cells treated with calcitriol and is represented in the tables.

We next sought to distinguish the global protein abundance differences between RCC and LCC when treated with calcitriol. DLD-1 and HCT 116 cells were seeded and grown for 48 h prior to treatment. Cells were treated with either 100 nM calcitriol dissolved in isopropanol or with the vehicle control isopropanol for 72 h to induce proteomic and metabolomic changes. This concentration was chosen based on previous studies of calcitriol treatment in colon and kidney cells [23,24]. To quantify the abundance changes in the proteome due to calcitriol treatment, we used isobaric tags for relative and absolute quantification (iTRAQ). Each biological condition was grown and treated in duplicate. Each sample was labeled with one of the eight iTRAQ tags at their *N*-terminus and *C*-terminal lysine residues (Figure 3A). Peptides were then fragmented during tandem mass spectrometry so that the iTRAQ tags would release their characteristic reporter that enables peptide quantification. Peptide reporter ion ratios were calculated and analyzed using various statistical methods to uncover proteins that were differentially regulated on each side of the colon after calcitriol treatment. 

We identified and quantified 6018 proteins at a 1% false discovery rate. Volcano plots extracted from the ProteoSign platform from the triplicate UPLC-MS/MS dataset plot the average change distribution between treated and untreated 2D cell culture. This data was plotted on a log2 scale on the x-axis and the calculated probability (*p*-value) on a −log10 scale on the y-axis (Figure 3B). Proteins that exceeded both threshold values—above line of 2.0 on the y-axis indicating statistical significance of *p* < 0.01 and beyond ±1.5 log2 fold change—were considered to be differentially regulated. Based on these stringent criteria, we found 56 proteins to be differentially regulated when DLD-1 cells were treated with calcitriol and 30 proteins to be differentially regulated when HCT 116 cells were treated with calcitriol (Figure 3C). 

Using the Ingenuity Pathway Analysis, we determined the canonical pathways that were differentially regulated on each side of the colon upon calcitriol treatment. The top five enriched pathways according to *p*-values are shown for each dataset (Figure 3D). Our results indicate that when DLD-1 cells, representing LCC, were treated with calcitriol, cholesterol biosynthesis was enriched. Of the top five pathways, four are involved in cholesterol biosynthesis. Based on the results for HCT 116 cells, representing RCC, the treatment with calcitriol enriched pathways distinct from LCC. For RCC, the pathways enriched include translation and amino acid biosynthesis and degradation. Interestingly, in RCC-derived cells treated with calcitriol, we also detected an enrichment of the granzyme B pathway, which is responsible for caspase-dependent apoptosis [25]. This data suggests that different pathways are enriched in RCC and LCC cells treated with calcitriol and implies that cancers deriving from the two separate sides of the colon should be studied separately.

Further examination of the proteins that were differentially regulated by calcitriol treatment in DLD-1 and HCT 116 cells reveals that only two proteins were altered in both cell lines. These two proteins are ribosomal protein L37 (RPL37) and protein S100-A10 (S100A10). There are 80 highly conserved human ribosomal proteins (RPs) that are necessary for protein synthesis inside the cell. It has been shown that RPs are often deregulated in cancer, including colon cancer [26]. RPL37 is reported to be overexpressed in colon cancer [27]. It has been suggested that the many phosphorylation sites found on RPL37 may be the substrate for a regulatory cascade of kinases that can be exploited by cancer for autonomous growth signaling and protein synthesis [27,28]. Our data show that when both LCC and RCC was treated with calcitriol, RPL37 was downregulated. 

S100A10 is a member of the S100 protein family that is involved in intracellular calcium binding [29]. S100A10 specifically binds to plasminogen, and plasminogen activation is a key step in tumor growth and invasion [30]. Additionally, S100A10 has been proposed as a prognostic marker for colorectal cancer as S100A10 overexpression is correlated with poor differentiation and increased mortality in colon cancer patients [19]. In our proteomic dataset, S100A10 was downregulated with calcitriol treatment in both RCC and LCC. Both of these proteins are overexpressed in colorectal cancer and were downregulated in RCC and LCC when treated with calcitriol. Interestingly, the fold change of downregulation for both proteins was more significant in DLD-1 cells, representing LCC. These data indicate that calcitriol treatment is an important mechanism influencing colorectal cancer development and can potentially be used in both LCC and RCC.

### 2.3. Untargeted Metabolomics to Determine Differentially Expressed Metabolites in LCC and RCC in Response to Treatment with Calcitriol

We next characterized the effect of calcitriol on RCC and LCC through analysis of the metabolome. Metabolomics is the study of physiologically relevant small molecules that are responsible for metabolic processes in organisms. The presence or quantity of these molecules can reflect the state of the cells, tissues, and organisms from which they are derived [31]. To look at metabolite abundance changes due to vitamin D treatment, DLD-1 and HCT 116 cells were grown for 48 h prior to treatment of 100 nM calcitriol dissolved in isopropanol or isopropanol for vehicle control for 72 h. Small molecules were then extracted and analyzed by LC-MS. In contrast to the mass spectrometry experiments to examine proteins, the conditions for these analyses were 100–900 *m*/*z* range with a mass resolution of 70,000 (at *m*/*z* 200). 

**Figure 4:**
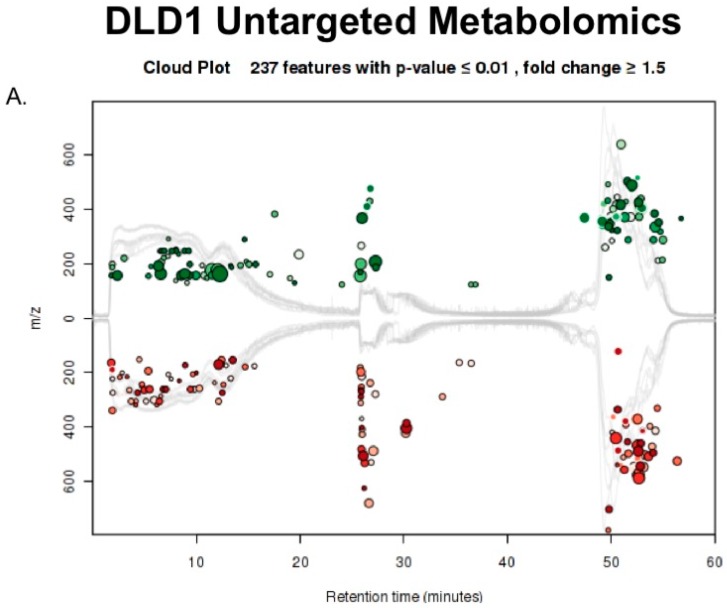

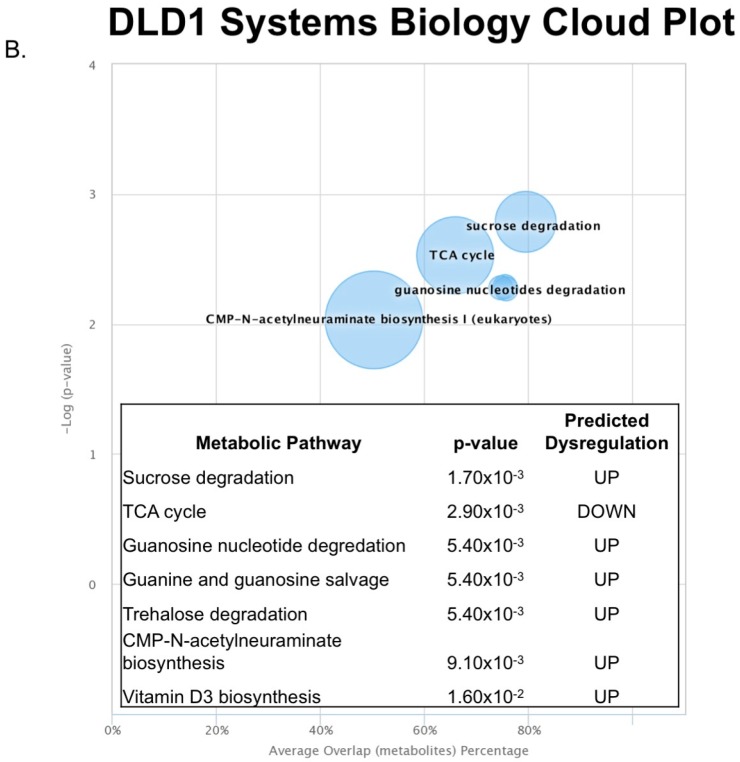
(**A**) XCMS online was used to compare metabolite differences between treated and untreated DLD-1 cells. A cloud plot was produced to compare features plotted on *m*/*z* (y-axis) and retention time (x-axis). XCMS identified 237 metabolites up or downregulated by treatment in DLD-1 cells, which are represented by green and red circles, respectively. Size and color of the circles correspond to the log fold change and *p*-value, respectively; (**B**) Using the 237 differential metabolites, XCMS maps the metabolites to metabolic pathways represented on a cloud plot. Pathways with greater percent overlap of metabolites and statistical significance will appear in the upper right corner of the cloud plot. Five pathways were found to have a *p*-value <0.01 and their predicted dysregulation is shown in the table.

Metabolomics data from technical triplicates of control or calcitriol-treated DLD-1 and HCT 116 cells were analyzed using XCMS Online. DLD-1 cells treated with calcitriol resulted in the identification of 14,335 features. XCMS extracted metabolomic features with statistically significant expression changes among the two groups to produce a list of differentially expressed features based on *p*-values (*p* ≤ 0.05, ≥1.5 fold change) [32]. Features were putatively identified using tandem mass spectra searched against the following databases: METLIN, Human Metabolome Database (HMD), and LIPIDMAPS. Using these conditions, 237 features were considered statistically different between the control and treated DLD-1 cells (Figure 4A). All of the 237 identified features with significant differential expression are listed in Supplemental Materials XLXS. These features were matched onto metabolic pathways to provide insight into the regulation arising from the calcitriol treatment. Using Fisher’s exact test, dysregulated metabolic pathways were identified by XCMS. With a *p*-value threshold of <0.01, our results demonstrate that DLD-1 cells treated with calcitriol responded with a putative downregulation of the TCA cycle and an upregulation of sucrose degradation, nucleotide degradation, and salvage pathways (Figure 4B).

**Figure 5:**
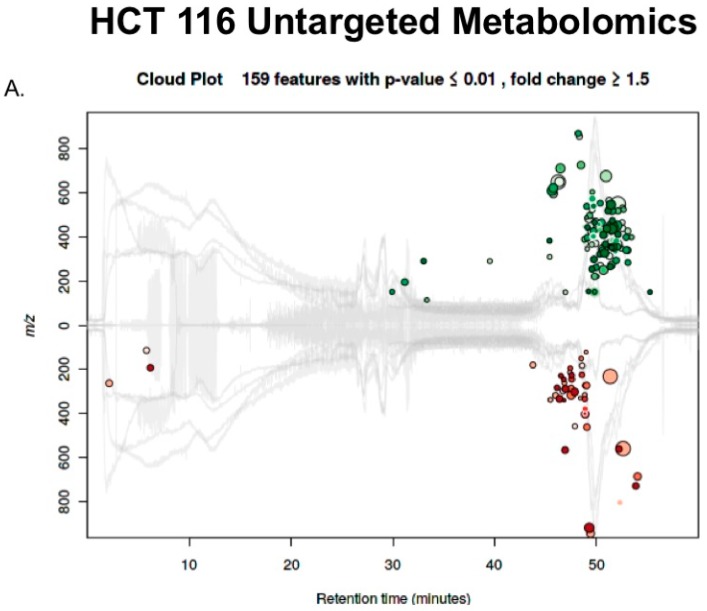

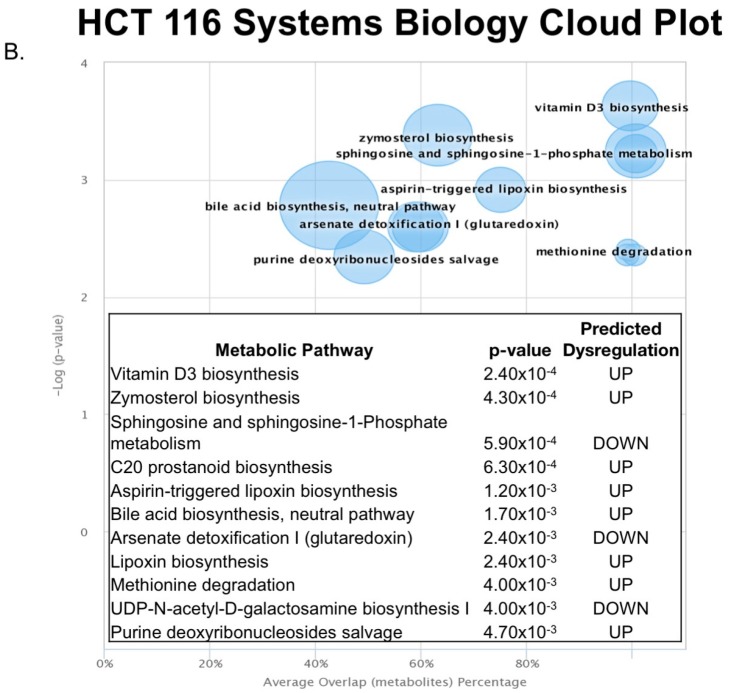
(**A**) XCMS online was used to compare metabolite differences between treated and untreated HCT 116 cells. A cloud plot was produced to compare features plotted on *m*/*z* (y-axis) and retention time (x-axis). XCMS identified 159 metabolites up or downregulated by treatment in HCT 116 cells, which are represented by green and red circles, respectively. Size and color of the circles correspond to the log fold change and *p*-value respectively; (**B**) Using the 159 differential metabolites, XCMS maps the metabolites to metabolic pathways represented on a cloud plot. Pathways with greater percent overlap of metabolites and statistical significance will appear in the upper right corner of the cloud plot. Eleven pathways were found to have a *p*-value < 0.01 and their predicted dysregulation is shown in the table.

These techniques were then used to compare the response to calcitriol treatment versus control in the HCT 116 cells. XCMS identified 14,472 features and categorized 159 to be differentially regulated (Figure 5A). These significantly different features were identified to be involved in many metabolic pathways (Figure 5B). Many of the pathways enriched are involved in cholesterol biosynthesis. The precursor for both cholesterol and calcitriol is 7-dehydrocholesterol (Figure 1). 

## 3. Discussion

In this study, we demonstrated that the proteomic and metabolomic changes that result from calcitriol treatment in LCC- and RCC-derived cells differ significantly. These results are in accordance with previous data suggesting that LCC and RCC should be studied as separate diseases. We successfully determined the original tumor location for multiple colon cell lines using PRAC expression. Utilizing this data, we compared calcitriol treatment in DLD-1 cells (representing LCC) and HCT 116 cells (representing RCC). Calcitriol treatment rendered very different proteomic and metabolomic changes in the LCC and RCC cells. Sporadic colon cancer is highly heterogeneous and is identified by many subtypes including microsatellite instability (MSI) status, flat versus polyploidy tumor shape, adenoma-carcinoma or serrated pathway, and the mutation status of KRAS, p53, and adenomatous polyposis coli (APC) [12,33,34]. Our results provide further evidence that tumor location should also be considered.

Vitamin D and its analogs have been shown to reduce colon cancer growth in mice xenografts [35,36]. Additionally, it is well established that vitamin D treatment has chemopreventative activity but the molecular mechanisms are largely unknown since vitamin D has broad transcriptional activity [7]. Calcitriol treatment in both RCC- and LCC-derived cells caused a decrease in two proteins that are pivotal in tumorigenesis: RPL37 and S100A10. Our data indicate that LCC-derived cells may be more responsive to vitamin D supplementation than RCC-derived cells, as demonstrated by the larger effect of calcitriol treatment on the proteome and metabolome. LCC-derived cells also showed a further decrease in RPL37 and S100A10 than RCC-derived cells. 

In addition, IPA has a function to predict upstream regulators based on the proteins that are found to be dysregulated by treatment. The only upstream regulator for both LCC and RCC predicted by IPA was N-Myc with an activation z-score in LCC of −0.849 and RCC of −0.391. N-Myc is a member of the Myc transcription factor family that acts as a proto-oncogene and has been reported to be overexpressed in colorectal cancer [37,38]. Interestingly, other members of the Myc family have been implicated as being responsible for the metabolic programing of colorectal cancer [39], and when this pathway is downregulated, cancer metabolism is altered [40]. Our data show that when RCC and LCC were treated with calcitriol, the Myc pathway was downregulated, thus presumably causing changes in metabolism in colorectal cancer. In support of this, our untargeted metabolomic analysis showed changes in metabolism. In LCC, we saw a predicted down regulation of the TCA cycle in addition to a predicted up regulation of nucleotide and sucrose degradation (Figure 4B). In RCC, we saw fewer pathways of metabolism being altered; instead, we observed pathways that are primarily involved in cholesterol biosynthesis (Figure 5B). 

Calcitriol supplementation has been reported to cause changes in cancer metabolism primarily through the reduction of Myc [39,40]. Our results from both proteomic and metabolomic studies of calcitriol treatment in LCC and RCC indicate that these changes in metabolic pathways are happening in colon cancer. This effect is likely more pronounced in LCC as it has a larger putative decrease of Myc.

RCC had less of a pronounced response to calcitriol treatment as metabolic pathways involved in metabolism were not dysregulated. However, metabolic pathways involved in inflammation and cholesterol biosynthesis were affected. Understanding the interplay between cholesterol and vitamin D supplementation is of ongoing interest, as it is known that statin drugs also inhibit vitamin D synthesis [41]. In addition to cholesterol, lipoxin biosynthesis was predicted to be upregulated. Lipoxin is a known anti-inflammatory agent, suggesting that calcitriol treatment plays a role in the anti-inflammation process, which is often dysregulated in colon cancer [42]. In this study, we have shown that calcitriol treatment caused different proteomic and metabolic signatures in LCC versus RCC, again providing support that the two diseases are distinct and must be studied separately. 

Overall, this evidence further implies that vitamin D is a valuable potential combatant for colon cancer patients. In the future, the optimal analog and concentration of vitamin D supplementation should be further studied. Randomized clinical trials should be conducted to determine the health benefits of vitamin D to right and left colon cancer treatment.

## 4. Materials and Methods 

### 4.1. Cell Culture Growth and Treatment

The human colon carcinoma cell lines HCT 116, HT-29, SW480, SW620, SW837, and DLD-1 were obtained from the American Type Culture Collection (ATCC) and grown in 2D cell culture using RPMI 1640 cell culture medium (Life Technologies, Calsbad, CA, USA) supplemented with 10% fetal bovine serum (FBS) (Thermo Scientific, Waltham, MA, USA). The provider assured authentication of these cell lines by cytogenetic analysis. In addition, the cell lines were validated by short tandem repeat (STR) analysis within the last 18 months. 

### 4.2. Microarray Dataset of Colon Cancer

For microarray data analysis, the Gene Expression Omnibus (http://www.ncbi.nlm.nih.gov/geo) data series GSE14333 was used [43]. This dataset is based on clinical colorectal cancer samples from the H. Lee Moffit Cancer Center in the United States and the Royal Melbourne Hospital, Western Hospital, and Peter MacCallum Cancer Center in Australia. The samples were hybridized to Affymetrix GeneChip^®^hgu133plus2 arrays. The authors computed expression values with cgrma with the CEL files and reported the survival endpoint in any relapse, distant, or local. 

### 4.3. RT-PCR PRAC Expression 

Total RNA was extracted from HCT 116, HT-29, SW480, SW620, SW837, and DLD-1 cells after 48 h of growth using the RNeasy Mini Kit according to the manufacturer’s protocol (Qiagen, Hilden, Germany). cDNA was generated using the High-Capacity cDNA Reverse Transcriptase kit (Applied Biosystems, Foster City, CA, USA) according to the manufacturer’s protocol. qRT-PCR was performed using StepOnePlus (Applied Biosystems) as previously described [44]. The primer sequences used were the following: hypoxanthine phosphoribosyltransferase 1 (HPRT1) Forward 5′-GCCATGAAGCAGGACTCTAAAGA-3′ and Reverse 5′-TTGGCATAACACAGCTGATTGAT-3′; prostate cancer susceptibility candidate (PRAC) Forward 5′-CCATTTCTCAGATCAAGGAC-3′ and Reverse 5′-GTCTCGCCCAGTAGATGTTT-3′. The comparative C_T_ method was used to quantify the transcripts relative to the housekeeping gene hypoxanthine phosphoribosyltransferase 1 (HPRT1). Samples were analyzed in triplicate and the median of each measurement was used for C_T_ quantitation.

### 4.4. Sample Preparation for Proteomic Analysis

Cells were lysed using a procedure previously described [44]. The Pierce bicinchoninic acid (BCA) protein assay kit (Thermo Scientific, Waltham, MA, USA) was used to quantify the total protein amount per sample. For all eight samples, 100 μg of protein was used for bottom-up proteomics protein digestion as previously described [44,45]. Peptides were desalted with 10 Oasis HLB Cartridges (Waters, Milford, MA, USA) and labeled (iTRAQ 8-plex reagents, AB Sciex, Farmington, MA, USA). Samples were next combined, dried under vacuum, and desalted again with 100 mg C18 Sep-Paks (Waters). The sample was then resuspended in 120 μL of 10 mM KH_2_PO_4_ in 20% ACN, pH 2.85 (Buffer A) for strong cation exchange (SCX) liquid chromatography fractionation. 

### 4.5. SCX Fractionation

To fractionate the iTRAQ labeled sample, we used SCX liquid chromatography. This was performed on an Alliance HPLC System (Waters). The protocol has been previously described by our lab [45]. 

### 4.6. Peptide Desalting

All 30 fractions were dried under vacuum and resuspended in 60 μL of 0.1% formic acid (FA) in water. The samples were then individually resuspended in 20 μL 0.1% FA in water and desalted (5 μg C18 ZipTip, ZTC18S096, Millipore, Burlington, MA, USA). Finally, peptides were dried under vacuum and resuspended in 10 μL of 0.1% FA in water for analysis by ultra-high performance liquid chromatography–electrospray tandem mass spectrometry (UPLC)-ESI-MS/MS.

### 4.7. UPLC-ESI-MS/MS Analysis

Using a nanoACQUITY UltraPerformance LC^®^ (UPLC^®^) (Waters), peptides were separated by injecting 2 μL of sample onto a C18 reversed-phase column (Waters, 100 μm × 100 mm, 1.7 μm particle, part No. 186003546, column temperature 40 °C). The mobile phases used were 0.1% FA in water and 0.1% FA in acetonitrile. The LC method was 75 minutes at a flow rate of 1 μL/min with the following gradient: 0–8 min at 2% buffer B, followed by a linear gradient of 2–6% buffer B for 2 min, 6–25% buffer B for 42 min, 25–50% buffer B for 6 min, 50–85% buffer B for 5 min, and maintained at 85% buffer B for 5 min. The column was equilibrated with 2% buffer B for 7 min. Separated peptides eluting from the column were then electrosprayed and analyzed by the mass spectrometer via electrospray ionization (ESI) and a Q-Exactive mass spectrometer (Thermo Fisher Scientific). The following parameters were used for the analysis: electrospray voltage was set to 2.2 kV, the ion transfer tube temperature was 280 °C, and the S-Lens RF level was 50.00. The data acquisition was programmed in data-dependent acquisition (DDA) mode with a top 12 method. Full MS scans were acquired in the Orbitrap mass analyzer with a 350–1800 *m*/*z* range with resolution at 70,000 1 microscan. The target value was 1.00 × 10^6^ and maximum injection time was 250 ms. Additionally, the isolation window was set as 1.0 *m*/*z* and the normalized collision energy at 31.0%. The tandem mass spectra were acquired in the Orbitrap mass analyzer with resolution 17,500 with the fixed first mass *m*/*z* 100.0. Similarly, the target value was 1.00 × 10^6^ and the number of microscans was set to 1, but the maximum injection time was 120 ms and the ion selection threshold was 5.0 × 10^6^ counts. Peptide match and exclude isotopes were turned on. Dynamic exclusion was set as 40 s. All injections were analyzed in triplicate. Raw data are available to the public on massIVE.

### 4.8. Proteomic Data Analysis

Raw data files were searched with MaxQuant version 1.5.6.0 with MASCOT version 2.2.4 and UniProt human and decoy databases. Parameters were set for trypsin with a maximum of two cleavage misses, precursor mass tolerance of 10–20 ppm, and fragment mass tolerance of 0.05 Da. Dynamic modifications included deamidation, oxidation, acetylation, and iTRAQ 8-plex labels on protein *N*-terminal and tyrosine. The only static modification was carbamidomethylation. A reverse sequence database was used to search the peptides to determine the false discovery rate (FDR) set to 0.01. iTRAQ 8-plex was used as the reporter ion quantitation type as well as instrument Orbitrap MS and MS/MS. Further data analysis was performed with ProteoSign, a web-based software for differential protein analysis [46]. Functional classification was performed using Ingenuity Pathway Analysis (IPA).

### 4.9. Small Molecule Extraction for Untargeted Metabolomics

After treatment with either calcitriol or isopropanol for vehicle control for 72 h, the cell culture medium was aspirated and the cells were washed with 2 mL PBS at 37 °C to remove the residual medium. Then, 1 mL of cold methanol/water (4:1) was added to the culture dish to quench the cells. The cells were then scraped, collected, and transferred to 1.5 mL centrifuge tubes. Cells were sonicated for three rounds of 1 min on and 30 sec off at 15% amplitude. Cells were kept on ice for the entire lysis. Cell debris was centrifuged at 4 °C for 10 min at 13,000× *g* and the supernatant was collected and dried. The residues were stored at −80 °C before analysis. For the UPLC-MS analysis of the metabolomics samples, the stored samples were resolved in 0.1% FA in water. 

### 4.10. Metabolomic UPLC-ESI-MS/MS Analysis

Full MS scans were acquired over the 100–900 *m*/*z* range with a mass resolution of 70,000 (at *m*/*z* 200). The AGC target value was 1.00 × 10^6^ and the ion selection threshold was 2.50 × 10^4^. The maximum injection time was 250 ms for full MS scans and 80 ms for tandem mass spectra. The dynamic exclusion time was set to 30 s. Raw data are available to the public on massIVE.

### 4.11. Data Analysis Workflow 

Raw files were uploaded and submitted to XCMS Online, which aligns the data and automatically integrates and extracts the peak intensities [32]. Feature selection produces a list of differentially expressed metabolites based on the *p*-value (*p* ≤ 0.01, ≥ 1.5 fold change). 

### 4.12. Statistical Analyses 

All experiments were performed in biological triplicate. Data were analyzed using unpaired Student’s *t*-test for comparison of means to detect differences compared with control, as well as Pearson correlation coefficient to measure linear correlation between two variables. Differences were considered to be statistically significant at * *p* < 0.01.

## Supplementary Materials

The following are available online at www.mdpi.com/2218-1989/8/1/5/s1, XLXS: MaxQuant Data Excel Sheet containing the raw data from Proteosign and XCMS Online for quantification of proteins using iTRAQ and metabolomics analysis, as well as the data analysis for determining up and downregulated proteins and metabolites in treatment compared to the untreated control.
